# {1′-Phenyl-1′,2′,5′,6′,7′,7a’-hexa­hydro­spiro­[indeno­[1,2-*b*]quinoxaline-11,3′-pyrrolizin]-2′-yl}(*p*-tol­yl)methanone

**DOI:** 10.1107/S1600536812031480

**Published:** 2012-07-18

**Authors:** T. Srinivasan, S. Suhitha, S. Purushothaman, R. Raghunathan, D. Velmurugan

**Affiliations:** a Centre of Advanced Study in Crystallography and Biophysics, University of Madras, Guindy Campus, Chennai 600 025, India; b Department of Organic Chemistry, University of Madras, Guindy Campus, Chennai 600 025, India

## Abstract

In the title compound, C_35_H_29_N_3_O, the quinoxaline and indene systems are essentially planar, with maximum deviations of 0.047 (2) and 0.032 (2) Å for C atoms, respectively. The quinoxaline system forms a dihedral angle of 4.75 (3)° with the indene system. The pyrrolizine system is folded. The substituted five-membered ring adopts an envelope conformation. In the other five-membered ring, one C atom is disordered with a site-occupancy ratio of 0.676 (12):0.324 (12). In the crystal, mol­ecules are linked *via* C—H⋯O hydrogen bonds involving the bifurcated carbonyl O atom.

## Related literature

For the uses of pyrrolidine and quinoxaline derivatives, see: Amal Raj *et al.* (2003 ▶); Zarranz *et al.* (2003 ▶). For a related structure, see: Gayathri *et al.* (2005 ▶).;
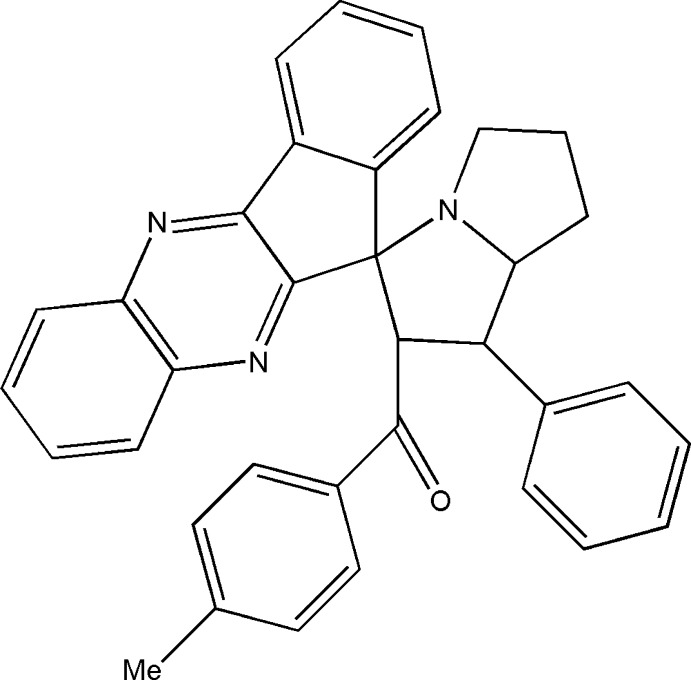


## Experimental

### 

#### Crystal data

C_35_H_29_N_3_O
*M* *_r_* = 507.61
Orthorhombic, 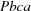

*a* = 16.4102 (6) Å
*b* = 16.4371 (6) Å
*c* = 20.0648 (7) Å
*V* = 5412.2 (3) Å^3^
*Z* = 8
Mo *K*α radiation
μ = 0.08 mm^−1^
*T* = 293 K
0.35 × 0.30 × 0.25 mm

#### Data collection

Bruker SMART APEXII area-detector diffractometer
47718 measured reflections
6643 independent reflections
3751 reflections with *I* > 2σ(*I*)
*R*_int_ = 0.044

#### Refinement

*R*[*F* ^2^ > 2σ(*F* ^2^)] = 0.049
*wR*(*F* ^2^) = 0.144
*S* = 1.01
6643 reflections
363 parameters
3 restraints
H-atom parameters constrained
Δρ_max_ = 0.26 e Å^−3^
Δρ_min_ = −0.25 e Å^−3^

### 

Data collection: *APEX2* (Bruker, 2008 ▶); cell refinement: *APEX2*; data reduction: *SAINT* (Bruker, 2008 ▶); program(s) used to solve structure: *SHELXS97* (Sheldrick, 2008 ▶); program(s) used to refine structure: *SHELXL97* (Sheldrick, 2008 ▶); molecular graphics: *ORTEP-3* (Farrugia, 1997 ▶); software used to prepare material for publication: *SHELXL97* and *PLATON* (Spek, 2009 ▶).

## Supplementary Material

Crystal structure: contains datablock(s) global, I. DOI: 10.1107/S1600536812031480/bt5959sup1.cif

Structure factors: contains datablock(s) I. DOI: 10.1107/S1600536812031480/bt5959Isup2.hkl

Supplementary material file. DOI: 10.1107/S1600536812031480/bt5959Isup3.cml

Additional supplementary materials:  crystallographic information; 3D view; checkCIF report

## Figures and Tables

**Table 1:** Hydrogen-bond geometry (Å, °)

*D*—H⋯*A*	*D*—H	H⋯*A*	*D*⋯*A*	*D*—H⋯*A*
C1—H1*C*⋯O1^i^	0.96	2.56	3.483 (2)	161
C22—H22⋯O1^ii^	0.93	2.48	3.361 (3)	158

Symmetry codes: (i) 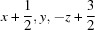; (ii) 
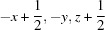
.

**Fig. 1.**
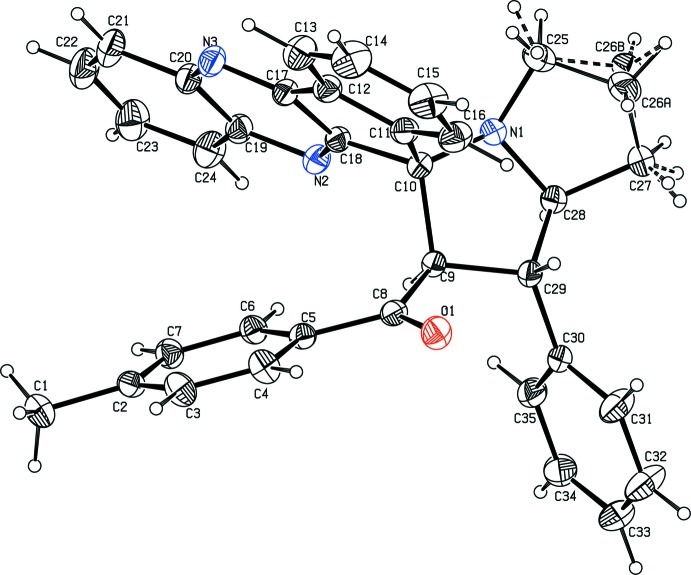
The molecular structure of the title compound, showing displacement ellipsoids drawn at the 30% probability level. H atoms are presented as a small spheres of arbitrary radius.

**Fig. 2.**
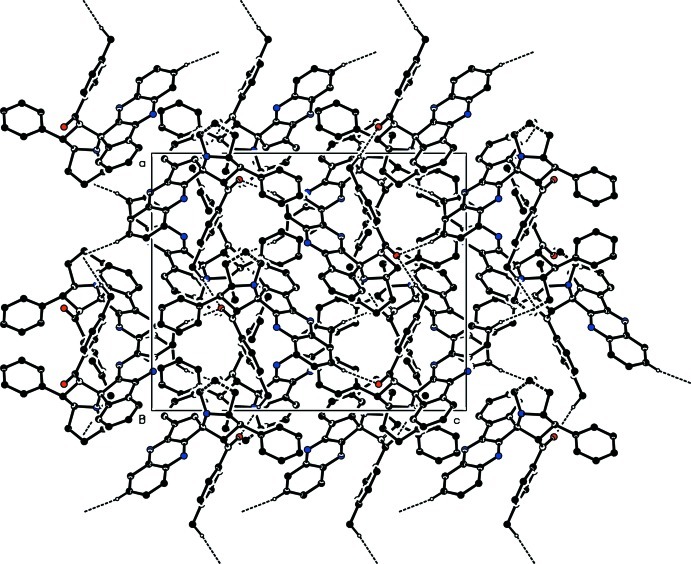
The crystal packing of the title compound viewed down a axis. H-atoms not involved in H-bonds have been excluded for clarity.

## supplementary crystallographic information

### Comment

Pyrrolidine derivatives are found to have anticonvulsant, antimicrobial and antifungal activities against various pathogens (Amal Raj *et al.*, 2003). Quinoxaline derivatives shown antibacterial, antiviral and anticancer properties (Zarranz *et al.*, 2003). As spiro pyrrolidine compounds are of great medicinal properties, we have undertaken the three dimensional structure of the title compound.

X-ray analysis confirms the molecular structure and atom connectivity of the compound as illustrated in Fig. 1. The quinoxaline moiety is essentially planar with a maximum deviation of -0.0472 (23) Å for C21 atom, and it forms the dihedral angles of 4.75 (3), 54.94 (5) and 29.70 (6) ° with the indene moiety, phenyl rings (C2–C7) and (C30–C35), respectively. The indene moiety is also essentially planar with a maximum deviation of 0.0320 (16) Å for C18 atom, and it forms the dihedral angles of 51.75 (6) and 32.41 (6) ° with the phenyl rings (C2–C7) and (C30–C35), respectively.

The pyrrolizine moiety is folded and twisted about the N—C bond common to the two five membered rings. The indene fused with quinoxaline, subsituted five membered ring adopts twisted conformation on C9 and C29 atoms with the puckering parameters of Q_2_ = 0.3846 (17) Å, φ_2_ = 95.8 (3) °. The unsubsituted five-membered ring has a disordered C atom with occupancy factor of 0.676 (12)/0.324 (12). The five-membered ring (N1/C25/C26A/C27/C28) adopts an envelope conformation on C26A for major occupancy atom with the puckering parameters Q_2_ = 0.309 (4) Å, φ_2_ = 77.9 (5) °. The five-membered ring (N1/C25/C26B/C27/C28) adopts an envelope conformation on C26B for minor occupancy atom with the puckering parameters Q_2_ = 0.238 (6) Å, φ_2_ = 247.0 (10) °. The title compound exhibits structural similarities with the already reported related structures (Gayathri *et al.*2005).

In the crystal packing, molecules are linked *via* bifurcated C—H···O intermolecular hydrogen bonds involving the carbonyl group O atom as a single acceptor (Table. 1). The packing view of the compound is shown in Fig. 2.

### Experimental

A mixture of ninhydrin (1 mmol) and 1, 2-phenylenediamine (1 mmol) was stirred for 10 min in 10 mL of toluene followed by the addition of L-proline (1 mmol) and a solution of (E)-3-phenyl-1-p-tolylprop-2-en-1-one (1 mmol) in 10 ml of toluene. The mixture was then refluxed until completion of the reaction as evidenced by TLC. The solvent was removed in vacuo and the crude product was subjected to column chromatography using petroleum ether/ethyl acetate (4:1) as eluent.

### Refinement

Hydrogen atoms were placed in calculated positions with C—H = 0.93 Å to 0.98 Å and refined using a riding model with fixed isotropic displacement parameters:*U*_iso_(H) = 1.5*U*_eq_(C) for methyl group and *U*_iso_(H) = 1.2*U*_eq_(C) for other groups. The distances between the disordered atoms and its neighbours were restrained to be equal with an effective e.s.d. of 0.01Å and the components of the ADP's of C25 and C26A in direction of the bond between them were restrained to be equal with an effective e.s.d. of 0.01Å^2^.

### Crystal data

**Table:**

C_35_H_29_N_3_O	*F*(000) = 2144
*M**_r_* = 507.61	*D*_x_ = 1.246 Mg m^−^^3^
Orthorhombic, *P**b**c**a*	Mo *K*α radiation, λ = 0.71073 Å
Hall symbol: -P 2ac 2ab	Cell parameters from 6643 reflections
*a* = 16.4102 (6) Å	θ = 2.0–28.3°
*b* = 16.4371 (6) Å	µ = 0.08 mm^−^^1^
*c* = 20.0648 (7) Å	*T* = 293 K
*V* = 5412.2 (3) Å^3^	Block, colourless
*Z* = 8	0.35 × 0.30 × 0.25 mm

### Data collection

**Table:**

Bruker SMART APEXII area-detector diffractometer	3751 reflections with *I* > 2σ(*I*)
Radiation source: fine-focus sealed tube	*R*_int_ = 0.044
Graphite monochromator	θ_max_ = 28.3°, θ_min_ = 2.0°
ω and φ scans	*h* = −18→21
47718 measured reflections	*k* = −21→21
6643 independent reflections	*l* = −25→26

### Refinement

**Table:**

Refinement on *F*^2^	Primary atom site location: structure-invariant direct methods
Least-squares matrix: full	Secondary atom site location: difference Fourier map
*R*[*F*^2^ > 2σ(*F*^2^)] = 0.049	Hydrogen site location: inferred from neighbouring sites
*wR*(*F*^2^) = 0.144	H-atom parameters constrained
*S* = 1.01	*w* = 1/[σ^2^(*F*_o_^2^) + (0.0614*P*)^2^ + 1.0312*P*] where *P* = (*F*_o_^2^ + 2*F*_c_^2^)/3
6643 reflections	(Δ/σ)_max_ < 0.001
363 parameters	Δρ_max_ = 0.26 e Å^−^^3^
3 restraints	Δρ_min_ = −0.25 e Å^−^^3^

### Special details

**Table:**

Geometry. All esds (except the esd in the dihedral angle between two l.s. planes) are estimated using the full covariance matrix. The cell esds are taken into account individually in the estimation of esds in distances, angles and torsion angles; correlations between esds in cell parameters are only used when they are defined by crystal symmetry. An approximate (isotropic) treatment of cell esds is used for estimating esds involving l.s. planes.
Refinement. Refinement of F^2^ against ALL reflections. The weighted R-factor wR and goodness of fit S are based on F^2^, conventional R-factors R are based on F, with F set to zero for negative F^2^. The threshold expression of F^2^ > 2sigma(F^2^) is used only for calculating R-factors(gt) etc. and is not relevant to the choice of reflections for refinement. R-factors based on F^2^ are statistically about twice as large as those based on F, and R- factors based on ALL data will be even larger.

### Fractional atomic coordinates and isotropic or equivalent isotropic displacement parameters (Å^2^)

**Table:**

	*x*	*y*	*z*	*U*_iso_*/*U*_eq_	Occ. (<1)
O1	0.10252 (8)	0.10058 (8)	0.72132 (6)	0.0618 (4)	
N1	0.01215 (8)	−0.11050 (9)	0.82474 (7)	0.0508 (4)	
N2	0.17619 (9)	−0.11583 (8)	0.89736 (7)	0.0494 (4)	
N3	0.15224 (9)	−0.00304 (10)	1.00457 (7)	0.0574 (4)	
C1	0.44254 (11)	0.18403 (13)	0.86999 (10)	0.0686 (6)	
H1A	0.4539	0.1586	0.9121	0.103*	
H1B	0.4368	0.2417	0.8762	0.103*	
H1C	0.4866	0.1736	0.8397	0.103*	
C2	0.36464 (10)	0.14989 (11)	0.84176 (8)	0.0506 (4)	
C3	0.30762 (11)	0.19957 (11)	0.81147 (10)	0.0619 (5)	
H3	0.3171	0.2553	0.8094	0.074*	
C4	0.23703 (12)	0.16835 (11)	0.78430 (10)	0.0588 (5)	
H4	0.2003	0.2032	0.7635	0.071*	
C5	0.21991 (10)	0.08581 (10)	0.78749 (7)	0.0432 (4)	
C6	0.27605 (10)	0.03572 (10)	0.81850 (8)	0.0481 (4)	
H6	0.2658	−0.0198	0.8217	0.058*	
C7	0.34726 (10)	0.06738 (11)	0.84484 (9)	0.0525 (4)	
H7	0.3844	0.0325	0.8651	0.063*	
C8	0.14256 (10)	0.05584 (10)	0.75697 (8)	0.0445 (4)	
C9	0.11326 (9)	−0.03001 (10)	0.77066 (7)	0.0413 (4)	
H9	0.1610	−0.0658	0.7723	0.050*	
C10	0.06490 (10)	−0.03986 (10)	0.83808 (7)	0.0436 (4)	
C11	0.02337 (10)	0.03781 (10)	0.86317 (8)	0.0482 (4)	
C12	0.05057 (11)	0.05879 (10)	0.92674 (8)	0.0503 (4)	
C13	0.02162 (13)	0.12754 (12)	0.95904 (10)	0.0685 (6)	
H13	0.0401	0.1411	1.0014	0.082*	
C14	−0.03536 (15)	0.17546 (13)	0.92672 (12)	0.0805 (7)	
H14	−0.0553	0.2220	0.9474	0.097*	
C15	−0.06280 (14)	0.15450 (14)	0.86396 (12)	0.0767 (6)	
H15	−0.1015	0.1870	0.8431	0.092*	
C16	−0.03374 (12)	0.08618 (13)	0.83169 (10)	0.0636 (5)	
H16	−0.0524	0.0729	0.7893	0.076*	
C17	0.11145 (10)	−0.00050 (10)	0.94844 (8)	0.0461 (4)	
C18	0.12229 (10)	−0.05761 (10)	0.89607 (7)	0.0425 (4)	
C19	0.22135 (10)	−0.11977 (10)	0.95527 (9)	0.0510 (4)	
C20	0.20808 (11)	−0.06546 (11)	1.00866 (8)	0.0547 (4)	
C21	0.25522 (14)	−0.07419 (14)	1.06687 (10)	0.0742 (6)	
H21	0.2462	−0.0398	1.1029	0.089*	
C22	0.31366 (15)	−0.13228 (15)	1.07069 (12)	0.0835 (7)	
H22	0.3440	−0.1376	1.1096	0.100*	
C23	0.32873 (14)	−0.18387 (14)	1.01731 (12)	0.0807 (7)	
H23	0.3703	−0.2222	1.0203	0.097*	
C24	0.28306 (13)	−0.17897 (12)	0.96040 (10)	0.0687 (6)	
H24	0.2927	−0.2146	0.9253	0.082*	
C25	−0.07181 (12)	−0.11437 (16)	0.84711 (11)	0.0787 (7)	
H25A	−0.0855	−0.0668	0.8734	0.094*	0.676 (12)
H25B	−0.0809	−0.1627	0.8739	0.094*	0.676 (12)
H25C	−0.0954	−0.0603	0.8484	0.094*	0.324 (12)
H25D	−0.0747	−0.1377	0.8915	0.094*	0.324 (12)
C26A	−0.1208 (2)	−0.1172 (5)	0.7858 (2)	0.0808 (16)	0.676 (12)
H26A	−0.1341	−0.0626	0.7710	0.097*	0.676 (12)
H26B	−0.1712	−0.1467	0.7934	0.097*	0.676 (12)
C26B	−0.1160 (5)	−0.1655 (8)	0.7995 (4)	0.065 (3)	0.324 (12)
H26C	−0.1713	−0.1460	0.7939	0.078*	0.324 (12)
H26D	−0.1179	−0.2214	0.8151	0.078*	0.324 (12)
C27	−0.06970 (12)	−0.15994 (16)	0.73483 (10)	0.0744 (6)	
H27A	−0.0827	−0.1415	0.6901	0.089*	0.676 (12)
H27B	−0.0770	−0.2184	0.7372	0.089*	0.676 (12)
H27C	−0.0938	−0.1193	0.7059	0.089*	0.324 (12)
H27D	−0.0698	−0.2119	0.7119	0.089*	0.324 (12)
C28	0.01680 (10)	−0.13568 (11)	0.75445 (8)	0.0510 (4)	
H28	0.0528	−0.1830	0.7505	0.061*	
C29	0.05461 (10)	−0.06337 (10)	0.71784 (7)	0.0441 (4)	
H29	0.0120	−0.0228	0.7099	0.053*	
C30	0.09388 (10)	−0.08434 (11)	0.65201 (8)	0.0471 (4)	
C31	0.06495 (14)	−0.05272 (14)	0.59322 (9)	0.0743 (6)	
H31	0.0208	−0.0173	0.5941	0.089*	
C32	0.09995 (17)	−0.07242 (17)	0.53289 (10)	0.0934 (8)	
H32	0.0789	−0.0504	0.4938	0.112*	
C33	0.16483 (15)	−0.12356 (15)	0.52992 (10)	0.0794 (7)	
H33	0.1885	−0.1365	0.4891	0.095*	
C34	0.19473 (13)	−0.15567 (14)	0.58740 (10)	0.0720 (6)	
H34	0.2392	−0.1908	0.5859	0.086*	
C35	0.15961 (11)	−0.13657 (13)	0.64785 (9)	0.0601 (5)	
H35	0.1806	−0.1593	0.6866	0.072*	

### Atomic displacement parameters (Å^2^)

**Table:**

	*U*^11^	*U*^22^	*U*^33^	*U*^12^	*U*^13^	*U*^23^
O1	0.0607 (8)	0.0626 (8)	0.0621 (8)	0.0001 (7)	−0.0130 (6)	0.0174 (6)
N1	0.0495 (8)	0.0587 (9)	0.0441 (8)	−0.0123 (7)	0.0010 (6)	−0.0024 (6)
N2	0.0563 (8)	0.0482 (8)	0.0436 (8)	0.0015 (7)	−0.0056 (7)	0.0016 (6)
N3	0.0650 (9)	0.0627 (9)	0.0447 (8)	−0.0033 (8)	−0.0066 (7)	−0.0078 (7)
C1	0.0559 (11)	0.0729 (13)	0.0769 (14)	−0.0180 (10)	−0.0028 (10)	−0.0010 (11)
C2	0.0476 (10)	0.0556 (10)	0.0487 (10)	−0.0081 (9)	0.0067 (8)	−0.0055 (8)
C3	0.0587 (11)	0.0429 (10)	0.0840 (14)	−0.0057 (9)	−0.0002 (10)	−0.0009 (9)
C4	0.0536 (11)	0.0450 (10)	0.0779 (13)	0.0023 (9)	−0.0038 (9)	0.0045 (9)
C5	0.0443 (9)	0.0432 (9)	0.0419 (9)	−0.0002 (7)	0.0024 (7)	−0.0004 (7)
C6	0.0491 (9)	0.0427 (9)	0.0525 (10)	−0.0036 (8)	−0.0005 (8)	0.0008 (7)
C7	0.0470 (9)	0.0557 (11)	0.0550 (10)	−0.0004 (9)	−0.0040 (8)	0.0035 (8)
C8	0.0456 (9)	0.0495 (9)	0.0386 (8)	0.0027 (8)	0.0013 (7)	0.0009 (7)
C9	0.0404 (8)	0.0464 (9)	0.0372 (8)	−0.0006 (7)	−0.0028 (7)	−0.0019 (7)
C10	0.0453 (9)	0.0482 (9)	0.0373 (8)	−0.0018 (8)	−0.0020 (7)	−0.0001 (7)
C11	0.0463 (9)	0.0534 (10)	0.0449 (9)	0.0019 (8)	0.0030 (7)	0.0026 (8)
C12	0.0538 (10)	0.0497 (10)	0.0474 (10)	−0.0022 (8)	0.0040 (8)	−0.0034 (8)
C13	0.0794 (14)	0.0608 (12)	0.0653 (13)	0.0071 (11)	0.0020 (11)	−0.0152 (10)
C14	0.0912 (16)	0.0629 (13)	0.0876 (16)	0.0209 (12)	0.0099 (13)	−0.0118 (12)
C15	0.0751 (14)	0.0733 (14)	0.0817 (15)	0.0268 (12)	0.0058 (12)	0.0067 (12)
C16	0.0630 (12)	0.0727 (13)	0.0549 (11)	0.0139 (10)	0.0014 (9)	0.0049 (10)
C17	0.0507 (9)	0.0486 (9)	0.0389 (9)	−0.0062 (8)	−0.0010 (7)	−0.0017 (7)
C18	0.0470 (9)	0.0428 (9)	0.0377 (8)	−0.0056 (8)	−0.0007 (7)	0.0010 (7)
C19	0.0546 (10)	0.0519 (10)	0.0466 (10)	−0.0043 (8)	−0.0071 (8)	0.0070 (8)
C20	0.0586 (11)	0.0604 (11)	0.0450 (10)	−0.0083 (9)	−0.0095 (8)	0.0022 (8)
C21	0.0857 (15)	0.0818 (15)	0.0552 (12)	−0.0032 (13)	−0.0243 (11)	−0.0026 (10)
C22	0.0901 (16)	0.0902 (17)	0.0701 (15)	−0.0043 (14)	−0.0345 (13)	0.0111 (13)
C23	0.0800 (15)	0.0773 (15)	0.0849 (16)	0.0115 (12)	−0.0256 (13)	0.0150 (13)
C24	0.0778 (14)	0.0635 (12)	0.0650 (13)	0.0102 (11)	−0.0148 (11)	0.0063 (10)
C25	0.0611 (12)	0.1042 (18)	0.0708 (14)	−0.0241 (12)	0.0196 (10)	−0.0172 (12)
C26A	0.0440 (18)	0.124 (4)	0.074 (2)	−0.007 (3)	−0.0019 (16)	0.019 (3)
C26B	0.047 (4)	0.090 (7)	0.059 (5)	−0.013 (5)	0.009 (3)	−0.009 (4)
C27	0.0597 (12)	0.1047 (17)	0.0589 (12)	−0.0316 (12)	−0.0032 (10)	−0.0059 (12)
C28	0.0487 (10)	0.0578 (10)	0.0467 (10)	−0.0079 (8)	0.0012 (8)	−0.0079 (8)
C29	0.0394 (8)	0.0534 (10)	0.0396 (8)	0.0007 (8)	−0.0036 (7)	−0.0028 (7)
C30	0.0451 (9)	0.0564 (10)	0.0397 (9)	−0.0064 (8)	−0.0020 (7)	−0.0064 (8)
C31	0.0838 (14)	0.0957 (16)	0.0433 (11)	0.0246 (13)	−0.0046 (10)	−0.0039 (10)
C32	0.121 (2)	0.119 (2)	0.0403 (11)	0.0281 (18)	−0.0017 (12)	−0.0009 (12)
C33	0.0903 (16)	0.1002 (18)	0.0477 (12)	−0.0034 (14)	0.0130 (11)	−0.0157 (11)
C34	0.0605 (12)	0.0898 (16)	0.0658 (13)	0.0040 (11)	0.0090 (10)	−0.0210 (12)
C35	0.0519 (10)	0.0790 (13)	0.0495 (10)	0.0037 (10)	0.0007 (8)	−0.0073 (9)

### Geometric parameters (Å, º)

**Table:**

O1—C8	1.2184 (19)	C19—C20	1.411 (2)
N1—C25	1.450 (2)	C20—C21	1.408 (2)
N1—C28	1.472 (2)	C21—C22	1.355 (3)
N1—C10	1.473 (2)	C21—H21	0.9300
N2—C18	1.303 (2)	C22—C23	1.388 (3)
N2—C19	1.380 (2)	C22—H22	0.9300
N3—C17	1.311 (2)	C23—C24	1.368 (3)
N3—C20	1.378 (2)	C23—H23	0.9300
C1—C2	1.507 (2)	C24—H24	0.9300
C1—H1A	0.9600	C25—C26B	1.464 (6)
C1—H1B	0.9600	C25—C26A	1.470 (4)
C1—H1C	0.9600	C25—H25A	0.9700
C2—C3	1.383 (3)	C25—H25B	0.9700
C2—C7	1.387 (2)	C25—H25C	0.9700
C3—C4	1.379 (3)	C25—H25D	0.9700
C3—H3	0.9300	C26A—C27	1.499 (4)
C4—C5	1.387 (2)	C26A—H26A	0.9700
C4—H4	0.9300	C26A—H26B	0.9700
C5—C6	1.383 (2)	C26B—C27	1.507 (6)
C5—C8	1.493 (2)	C26B—H26C	0.9700
C6—C7	1.384 (2)	C26B—H26D	0.9700
C6—H6	0.9300	C27—C28	1.526 (2)
C7—H7	0.9300	C27—H27A	0.9700
C8—C9	1.516 (2)	C27—H27B	0.9700
C9—C29	1.533 (2)	C27—H27C	0.9700
C9—C10	1.577 (2)	C27—H27D	0.9700
C9—H9	0.9800	C28—C29	1.529 (2)
C10—C18	1.525 (2)	C28—H28	0.9800
C10—C11	1.532 (2)	C29—C30	1.509 (2)
C11—C16	1.382 (2)	C29—H29	0.9800
C11—C12	1.395 (2)	C30—C31	1.374 (2)
C12—C13	1.386 (2)	C30—C35	1.381 (2)
C12—C17	1.462 (2)	C31—C32	1.378 (3)
C13—C14	1.384 (3)	C31—H31	0.9300
C13—H13	0.9300	C32—C33	1.358 (3)
C14—C15	1.381 (3)	C32—H32	0.9300
C14—H14	0.9300	C33—C34	1.360 (3)
C15—C16	1.381 (3)	C33—H33	0.9300
C15—H15	0.9300	C34—C35	1.379 (3)
C16—H16	0.9300	C34—H34	0.9300
C17—C18	1.420 (2)	C35—H35	0.9300
C19—C24	1.408 (3)		
			
C25—N1—C28	109.48 (14)	C22—C23—H23	119.7
C25—N1—C10	122.45 (15)	C23—C24—C19	119.7 (2)
C28—N1—C10	111.43 (12)	C23—C24—H24	120.1
C18—N2—C19	114.56 (14)	C19—C24—H24	120.1
C17—N3—C20	114.49 (15)	N1—C25—C26B	107.1 (3)
C2—C1—H1A	109.5	N1—C25—C26A	105.2 (2)
C2—C1—H1B	109.5	N1—C25—H25A	110.7
H1A—C1—H1B	109.5	C26B—C25—H25A	134.7
C2—C1—H1C	109.5	C26A—C25—H25A	110.7
H1A—C1—H1C	109.5	N1—C25—H25B	110.7
H1B—C1—H1C	109.5	C26B—C25—H25B	79.3
C3—C2—C7	117.26 (16)	C26A—C25—H25B	110.7
C3—C2—C1	121.29 (17)	H25A—C25—H25B	108.8
C7—C2—C1	121.45 (17)	N1—C25—H25C	110.3
C4—C3—C2	121.49 (17)	C26B—C25—H25C	110.3
C4—C3—H3	119.3	C26A—C25—H25C	80.4
C2—C3—H3	119.3	H25B—C25—H25C	132.4
C3—C4—C5	121.05 (17)	N1—C25—H25D	110.3
C3—C4—H4	119.5	C26B—C25—H25D	110.3
C5—C4—H4	119.5	C26A—C25—H25D	136.8
C6—C5—C4	117.91 (16)	H25A—C25—H25D	78.9
C6—C5—C8	123.66 (15)	H25C—C25—H25D	108.6
C4—C5—C8	118.43 (15)	C25—C26A—C27	106.2 (3)
C5—C6—C7	120.68 (16)	C25—C26A—H26A	110.5
C5—C6—H6	119.7	C27—C26A—H26A	110.5
C7—C6—H6	119.7	C25—C26A—H26B	110.5
C6—C7—C2	121.61 (17)	C27—C26A—H26B	110.5
C6—C7—H7	119.2	H26A—C26A—H26B	108.7
C2—C7—H7	119.2	C25—C26B—C27	106.1 (4)
O1—C8—C5	120.02 (15)	C25—C26B—H26C	110.5
O1—C8—C9	119.81 (15)	C27—C26B—H26C	110.5
C5—C8—C9	120.18 (14)	C25—C26B—H26D	110.5
C8—C9—C29	114.01 (13)	C27—C26B—H26D	110.5
C8—C9—C10	114.25 (12)	H26C—C26B—H26D	108.7
C29—C9—C10	103.91 (12)	C26A—C27—C28	102.8 (2)
C8—C9—H9	108.1	C26B—C27—C28	105.2 (3)
C29—C9—H9	108.1	C26A—C27—H27A	111.2
C10—C9—H9	108.1	C26B—C27—H27A	134.8
N1—C10—C18	110.53 (13)	C28—C27—H27A	111.2
N1—C10—C11	117.08 (13)	C26A—C27—H27B	111.2
C18—C10—C11	100.57 (12)	C26B—C27—H27B	80.5
N1—C10—C9	102.76 (12)	C28—C27—H27B	111.2
C18—C10—C9	111.31 (12)	H27A—C27—H27B	109.1
C11—C10—C9	114.85 (13)	C26A—C27—H27C	81.8
C16—C11—C12	119.52 (17)	C26B—C27—H27C	110.7
C16—C11—C10	129.12 (16)	C28—C27—H27C	110.7
C12—C11—C10	111.36 (14)	H27B—C27—H27C	131.5
C13—C12—C11	121.29 (17)	C26A—C27—H27D	137.4
C13—C12—C17	129.65 (17)	C26B—C27—H27D	110.7
C11—C12—C17	109.04 (15)	C28—C27—H27D	110.7
C14—C13—C12	118.4 (2)	H27A—C27—H27D	80.6
C14—C13—H13	120.8	H27C—C27—H27D	108.8
C12—C13—H13	120.8	N1—C28—C27	105.81 (14)
C15—C14—C13	120.4 (2)	N1—C28—C29	105.24 (13)
C15—C14—H14	119.8	C27—C28—C29	117.17 (16)
C13—C14—H14	119.8	N1—C28—H28	109.4
C14—C15—C16	121.2 (2)	C27—C28—H28	109.4
C14—C15—H15	119.4	C29—C28—H28	109.4
C16—C15—H15	119.4	C30—C29—C28	114.60 (14)
C15—C16—C11	119.19 (19)	C30—C29—C9	114.75 (13)
C15—C16—H16	120.4	C28—C29—C9	101.58 (12)
C11—C16—H16	120.4	C30—C29—H29	108.5
N3—C17—C18	123.40 (16)	C28—C29—H29	108.5
N3—C17—C12	128.77 (15)	C9—C29—H29	108.5
C18—C17—C12	107.82 (14)	C31—C30—C35	116.95 (16)
N2—C18—C17	123.76 (14)	C31—C30—C29	121.17 (16)
N2—C18—C10	125.07 (14)	C35—C30—C29	121.88 (15)
C17—C18—C10	111.13 (14)	C30—C31—C32	121.4 (2)
N2—C19—C24	118.70 (16)	C30—C31—H31	119.3
N2—C19—C20	121.78 (16)	C32—C31—H31	119.3
C24—C19—C20	119.51 (16)	C33—C32—C31	120.7 (2)
N3—C20—C21	119.38 (17)	C33—C32—H32	119.6
N3—C20—C19	121.89 (15)	C31—C32—H32	119.6
C21—C20—C19	118.70 (18)	C32—C33—C34	119.07 (19)
C22—C21—C20	120.5 (2)	C32—C33—H33	120.5
C22—C21—H21	119.7	C34—C33—H33	120.5
C20—C21—H21	119.7	C33—C34—C35	120.5 (2)
C21—C22—C23	120.8 (2)	C33—C34—H34	119.8
C21—C22—H22	119.6	C35—C34—H34	119.8
C23—C22—H22	119.6	C34—C35—C30	121.40 (18)
C24—C23—C22	120.7 (2)	C34—C35—H35	119.3
C24—C23—H23	119.7	C30—C35—H35	119.3
			
C7—C2—C3—C4	−1.2 (3)	C11—C10—C18—N2	−175.19 (15)
C1—C2—C3—C4	178.60 (18)	C9—C10—C18—N2	−53.1 (2)
C2—C3—C4—C5	1.2 (3)	N1—C10—C18—C17	−121.43 (14)
C3—C4—C5—C6	−0.3 (3)	C11—C10—C18—C17	2.92 (16)
C3—C4—C5—C8	−179.86 (17)	C9—C10—C18—C17	125.04 (14)
C4—C5—C6—C7	−0.6 (2)	C18—N2—C19—C24	−177.85 (16)
C8—C5—C6—C7	178.94 (15)	C18—N2—C19—C20	1.2 (2)
C5—C6—C7—C2	0.6 (3)	C17—N3—C20—C21	−179.83 (17)
C3—C2—C7—C6	0.3 (3)	C17—N3—C20—C19	2.1 (2)
C1—C2—C7—C6	−179.50 (17)	N2—C19—C20—N3	−3.4 (3)
C6—C5—C8—O1	−168.12 (16)	C24—C19—C20—N3	175.65 (17)
C4—C5—C8—O1	11.4 (2)	N2—C19—C20—C21	178.59 (17)
C6—C5—C8—C9	12.4 (2)	C24—C19—C20—C21	−2.4 (3)
C4—C5—C8—C9	−168.10 (15)	N3—C20—C21—C22	−176.3 (2)
O1—C8—C9—C29	22.8 (2)	C19—C20—C21—C22	1.8 (3)
C5—C8—C9—C29	−157.73 (13)	C20—C21—C22—C23	0.5 (4)
O1—C8—C9—C10	−96.54 (17)	C21—C22—C23—C24	−2.2 (4)
C5—C8—C9—C10	82.97 (17)	C22—C23—C24—C19	1.5 (3)
C25—N1—C10—C18	101.23 (19)	N2—C19—C24—C23	179.85 (19)
C28—N1—C10—C18	−126.38 (14)	C20—C19—C24—C23	0.8 (3)
C25—N1—C10—C11	−13.1 (2)	C28—N1—C25—C26B	17.7 (7)
C28—N1—C10—C11	119.32 (15)	C10—N1—C25—C26B	150.9 (7)
C25—N1—C10—C9	−139.91 (17)	C28—N1—C25—C26A	−17.1 (4)
C28—N1—C10—C9	−7.53 (16)	C10—N1—C25—C26A	116.0 (4)
C8—C9—C10—N1	153.63 (13)	N1—C25—C26A—C27	30.8 (6)
C29—C9—C10—N1	28.79 (15)	C26B—C25—C26A—C27	−67.3 (6)
C8—C9—C10—C18	−88.06 (16)	N1—C25—C26B—C27	−25.4 (10)
C29—C9—C10—C18	147.10 (13)	C26A—C25—C26B—C27	66.4 (6)
C8—C9—C10—C11	25.37 (18)	C25—C26A—C27—C26B	66.4 (5)
C29—C9—C10—C11	−99.47 (15)	C25—C26A—C27—C28	−31.9 (5)
N1—C10—C11—C16	−62.9 (2)	C25—C26B—C27—C26A	−66.8 (6)
C18—C10—C11—C16	177.41 (17)	C25—C26B—C27—C28	23.3 (10)
C9—C10—C11—C16	57.8 (2)	C25—N1—C28—C27	−2.8 (2)
N1—C10—C11—C12	117.55 (16)	C10—N1—C28—C27	−141.40 (16)
C18—C10—C11—C12	−2.19 (17)	C25—N1—C28—C29	121.90 (17)
C9—C10—C11—C12	−121.78 (15)	C10—N1—C28—C29	−16.71 (17)
C16—C11—C12—C13	−0.2 (3)	C26A—C27—C28—N1	21.0 (4)
C10—C11—C12—C13	179.43 (16)	C26B—C27—C28—N1	−12.6 (6)
C16—C11—C12—C17	−178.88 (16)	C26A—C27—C28—C29	−95.9 (3)
C10—C11—C12—C17	0.76 (19)	C26B—C27—C28—C29	−129.5 (6)
C11—C12—C13—C14	0.0 (3)	N1—C28—C29—C30	158.35 (13)
C17—C12—C13—C14	178.36 (18)	C27—C28—C29—C30	−84.43 (19)
C12—C13—C14—C15	0.4 (3)	N1—C28—C29—C9	34.03 (16)
C13—C14—C15—C16	−0.7 (4)	C27—C28—C29—C9	151.25 (16)
C14—C15—C16—C11	0.5 (3)	C8—C9—C29—C30	72.33 (18)
C12—C11—C16—C15	0.0 (3)	C10—C9—C29—C30	−162.67 (14)
C10—C11—C16—C15	−179.59 (18)	C8—C9—C29—C28	−163.45 (13)
C20—N3—C17—C18	0.9 (2)	C10—C9—C29—C28	−38.46 (15)
C20—N3—C17—C12	−177.45 (16)	C28—C29—C30—C31	116.83 (19)
C13—C12—C17—N3	1.2 (3)	C9—C29—C30—C31	−126.17 (19)
C11—C12—C17—N3	179.73 (17)	C28—C29—C30—C35	−62.5 (2)
C13—C12—C17—C18	−177.35 (18)	C9—C29—C30—C35	54.5 (2)
C11—C12—C17—C18	1.17 (19)	C35—C30—C31—C32	0.1 (3)
C19—N2—C18—C17	1.9 (2)	C29—C30—C31—C32	−179.2 (2)
C19—N2—C18—C10	179.80 (14)	C30—C31—C32—C33	−0.5 (4)
N3—C17—C18—N2	−3.2 (3)	C31—C32—C33—C34	0.4 (4)
C12—C17—C18—N2	175.48 (15)	C32—C33—C34—C35	0.0 (4)
N3—C17—C18—C10	178.68 (15)	C33—C34—C35—C30	−0.4 (3)
C12—C17—C18—C10	−2.67 (18)	C31—C30—C35—C34	0.3 (3)
N1—C10—C18—N2	60.5 (2)	C29—C30—C35—C34	179.62 (17)

### Hydrogen-bond geometry (Å, º)

**Table:**

*D*—H···*A*	*D*—H	H···*A*	*D*···*A*	*D*—H···*A*
C1—H1*C*···O1^i^	0.96	2.56	3.483 (2)	161
C22—H22···O1^ii^	0.93	2.48	3.361 (3)	158

Symmetry codes: (i) *x*+1/2, *y*, −*z*+3/2; (ii) −*x*+1/2, −*y*, *z*+1/2.

## References

[bb1] Amal Raj, A., Raghunathan, R., Sridevi Kumari, M. R. & Raman, N. (2003). *Bioorg. Med. Chem.* **11**, 407–409. 10.1016/s0968-0896(02)00439-x 12517436

[bb2] Bruker (2008). *APEX2* and *SAINT* Bruker AXS Inc., Madison, Wisconsin, USA.

[bb3] Farrugia, L. J. (1997). *J. Appl. Cryst.* **30**, 565.

[bb4] Gayathri, D., Aravindan, P. G., Velmurugan, D., Ravikumar, K. & Sureshbabu, A. R. (2005). *Acta Cryst.* E**61**, o3124–o3126.

[bb5] Sheldrick, G. M. (2008). *Acta Cryst.* A**64**, 112–122. 10.1107/S0108767307043930 18156677

[bb6] Spek, A. L. (2009). *Acta Cryst.* D**65**, 148–155. 10.1107/S090744490804362X PMC2631630 19171970

[bb7] Zarranz, B., Jago, A., Aldana, I. & Monge, A. (2003). *Bioorg. Med. Chem.* **11**, 2149–2156. 10.1016/s0968-0896(03)00119-6 12713824

